# Establishment of a novel mouse model of adenomyosis suitable for longitudinal and quantitative analysis and perinatal outcome studies

**DOI:** 10.1038/s41598-022-22413-8

**Published:** 2022-10-20

**Authors:** Mohammed Elsherbini, Kaori Koga, Takehiro Hiraoka, Keiichi Kumasawa, Eiko Maki, Erina Satake, Ayumi Taguchi, Tomoko Makabe, Arisa Takeuchi, Gentaro Izumi, Masashi Takamura, Miyuki Harada, Tetsuya Hirata, Yasushi Hirota, Osamu Wada-Hiraike, Yutaka Osuga

**Affiliations:** 1 Department of Obstetrics and Gynecology, Faculty of Medicine, The University of Tokyo, 7-3-1, Hongo, Bunkyo, Tokyo, 113-8655 Japan; 2 Department of Obstetrics and Gynecology, Saitama Medical University, 38 Morohongo, Moroyama, Iruma, Saitama, 350-0495 Japan; 3 Department of Integrated Women’s Health, St Luke’s International Hospital, 9-1 Akashi, Chuo, Tokyo, 104-8560 Japan

**Keywords:** Endocrine reproductive disorders, Animal disease models, Experimental models of disease, Diseases, Medical research

## Abstract

The purpose of this study was to establish a novel mouse model of adenomyosis suitable for longitudinal and quantitative analyses and perinatal outcome studies. Using a 30 G needle, the entire uterine wall of one horn was mechanically punctured at a frequency of 100 times/1 cm (adenomyosis horn). The other horn was left unpunctured (control horn). Balb/c mice were sacrificed on day 14 (D14) or day 65 (D65) (n = 3 each). The uterus was fixed, paraffin-embedded, sliced, and stained. Lesions were detected and counted, and their volumes were measured. Cell proliferation and fibrosis were assessed by Ki67 and Masson’s Trichrome staining, respectively. Blood vessels were detected using CD31 immunostaining. Some of the mice (n = 4), were mated and the date of delivery, litter size, number of implantations, and number and volume of postpartum lesions were measured. The number of lesions per horn did not differ between D14 and D65. The volume of the entire lesion was significantly greater on D65 than on D14 (p < 0.0001). The volume of the epithelial part of the lesion was significantly greater in D65 (p < 0.0001). The volume of the stromal part of the lesion was also greater on D65 (p < 0.0001). The percentage of Ki67 positive cells in the epithelial part of the lesion was significantly higher on D14 (p < 0.05). In contrast, the percentage of Ki67-positive cells in the stromal part was significantly higher on D65 (p < 0.01). Vascular density in the lesions was higher in on D65 (p < 0.05). The percentage of fibrotic area was significantly higher on D65 (p < 0.01). The date of delivery was slightly earlier than that reported for healthy mice of the same strain. The litter size was smaller than that reported in previous research. The number of implantation sites did not differ between the control and the adenomyosis horn. The number and volume of lesions did not differ between the non-pregnant and postpartum groups. This model can be applied to evaluate the pathogenesis of adenomyosis, validate the efficacy of therapeutic agents, and evaluate the effect of adenomyosis on pregnancy and vice versa.

## Introduction

Adenomyosis is a benign gynecological condition characterized by the presence of endometrium-like epithelial and stromal tissues in the myometrium. Despite the prevalence and severity of symptoms such as pain and heavy menstrual bleeding (HMB), its pathogenesis and etiology have not yet been elucidated^1–4^. It has been proposed that the cause of adenomyosis is an invasion of the endometrium into the myometrium via a trauma of the endometrial-myometrial interface caused by delivery or intrauterine surgery, or hyperestrogenism with subsequent uterine hyperperistalsis^1,5^; however, there has been no experimental validation of this theory.

Recently, with the development of noninvasive diagnostic methods such as MRI, adenomyosis can now be diagnosed without a hysterectomy, and it has been revealed that this disease can cause pain, HMB, and infertility^2,6–8^. Patients with adenomyosis have also been found to have an increased risk of adverse perinatal events such as preterm delivery, fetal growth restriction, and preeclampsia^9–12^. The mechanisms by which adenomyosis causes perinatal adverse events have not yet been clarified, and no experimental methods have been established to verify them.

Animal models are useful in the study of adenomyosis to elucidate its pathogenesis and to develop therapies. To date, several laboratories have established mouse models of adenomyosis^13–19^. However, most models are not suitable for longitudinal studies because of the long time between lesion induction and establishment and the lack of knowledge regarding long-term lesion maintenance^20^. In addition, many endocrinologically induced adenomyosis models, such as the pituitary isograft mouse model^13^, prolonged progesterone exposure^14^ and prolonged tamoxifen exposure^15–19^ are not suitable for pregnancy studies because they require hormonal modification or oophorectomy.

The purpose of this study was to establish a novel mouse model of adenomyosis that was suitable for longitudinal and quantitative analyses and perinatal outcome studies. To create this model, we attempted to mimic the mechanical damage of the endometrium, which is generally considered a main cause of adenomyosis, in the mouse uterus.

## Methods

### Induction of adenomyosis by mechanical stimulation of the mouse uterus

**Figure 1:**
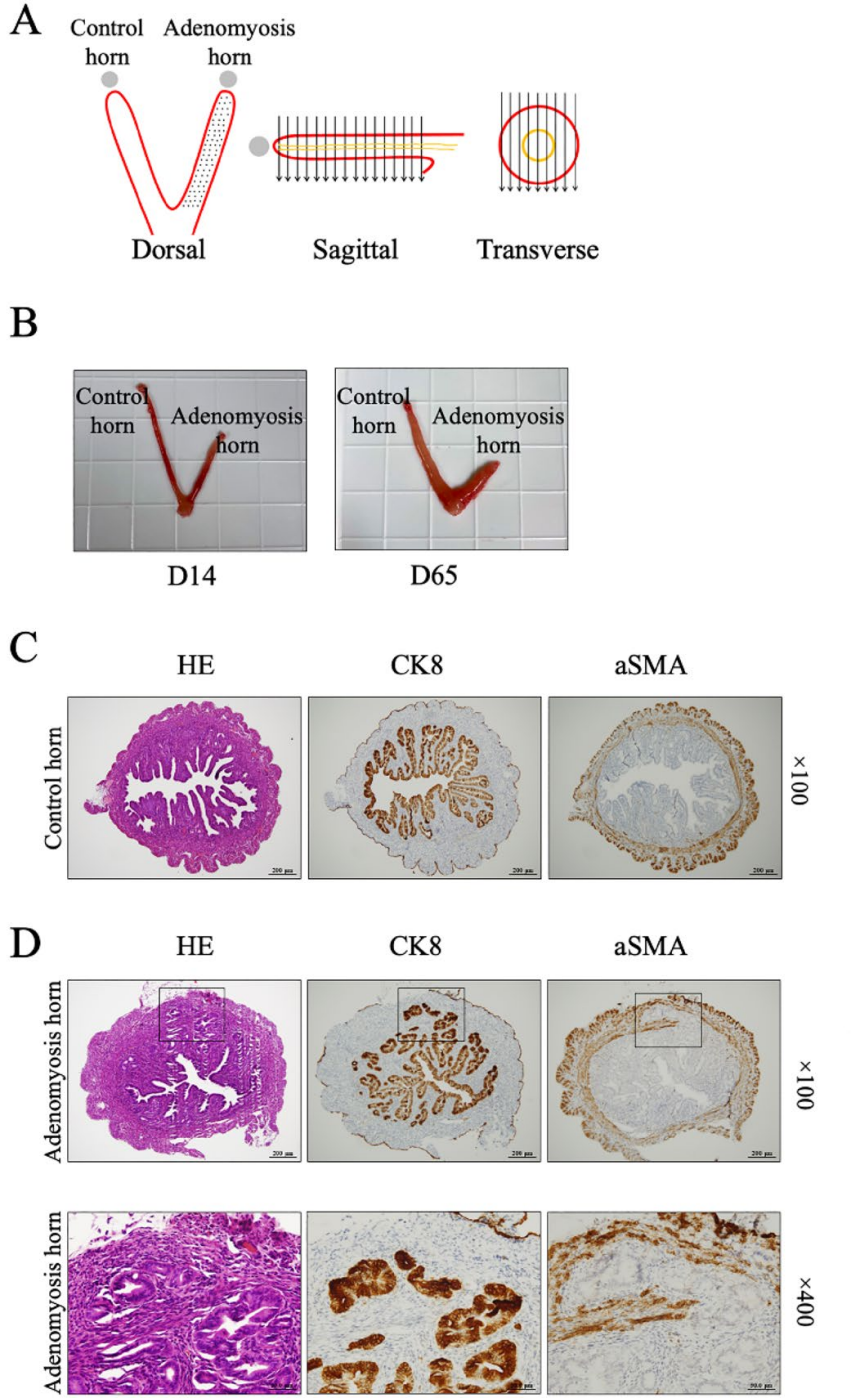
(A) A schema showing the punctures of the uterine horn. The entire uterine wall of one horn was mechanically punctured at a frequency of 100 times/1 cm. The puncture was crossed the whole uterine wall thickness. (**B)** A macroscopic finding of the uterus (D14 and D65). At D14, the adenomyosis horn was slightly shorter than the control horn, and the difference became more pronounced in D65. (**C**) A representative microscopic image of the control horn (D65, × 100). Hematoxylin and eosin staining (HE) shows the normal uterine architecture; cytokeratin 8 (CK8) shows eutopic endometrium and alpha smooth muscle actin (α SMA) shows smooth muscle architecture. (**D**) A representative microscopic image of the adenomyosis horn (D65, upper figures; ×100) (lower figures; ×400). Hematoxylin and eosin staining (HE) shows the uterine structure with an adenomyosis lesion (black box); cytokeratin 8 (CK8) shows eutopic endometrial epithelium and epithelium of the lesion (marked with black box), alpha smooth muscle actin (α SMA) shows smooth muscle structure that encircles adenomyosis lesion (marked with black box).

All procedures described in this study were conducted in accordance with the guidelines and regulations of the Animal Care and Use of the University of Tokyo Committee, and followed the recommendations in the ARRIVE guidelines. Six-week-old BALB/c female mice were purchased from Japan SLC, Inc. (Tokyo, Japan). The mice were fed a mouse diet and water and maintained on a light/dark cycle (12 h/12 h) under controlled living conditions. The adenomyosis mouse model was established as follows. First, a midline incision was made in the abdomen to expose the uterine horns. Using a 30 G (½ in.) needle, the entire uterine wall of one horn was mechanically punctured at a frequency of 100 times/1 cm (adenomyosis horn). The other horn was left unpunctured (control horn). The puncture penetrated through the entire uterine wall, as shown in Fig. 1A. The mice were sacrificed 14 (D14) and 65 days (D65) after the operation, and uteri were collected (n = 3 for each time point).

### Tissue preparation for hematoxylin and eosin staining (H&E staining)

Uterine tissues were sectioned, fixed in 10% formalin, dehydrated gradually in ethanol, and embedded in paraffin at 60 °C. For hematoxylin and eosin staining (H&E staining), sections were cut horizontally at a thickness of 6 μm and mounted on slides for analysis.

### Immunohistochemistry (IHC)

We investigated the extent of the adenomyosis lesions by cutting through the entire horn and quantitatively analyzing the number and volume of lesions. To confirm the presence of lesions, we prepared serial sections and performed immunohistochemistry. One section was stained with cytokeratin 8 (CK8) to detect epithelial cells and the other section with alpha smooth muscle actin (αSMA) to detect smooth muscle cells. The area was considered an adenomyosis lesion when epithelial cells were found within a smooth muscle layer. IHC was conducted as follows. All sections were deparaffinized in xylene and hydrated in a series of graded alcohols (100%, 100%, 100%, 90%, 80%, and 70%). Antigen retrieval was performed by boiling the samples in an ethylenediaminetetraacetic acid (EDTA) antigen retrieval solution (pH 9) (DAKO, Cat. No. K8004, Glostrup, Denmark) for 45 min at 98 °C. The samples were then incubated at room temperature for 30 min. The samples were washed three times in phosphate-buffered saline (PBS) and incubated with a peroxidase blocking agent (DAKO, Cat. No. S2023) for 10 min. The samples were washed again and incubated overnight with the diluted primary antibodies listed in Table 1 at 4 °C. The samples were then washed with PBS and incubated with the secondary antibody from the REAL EnVision Detection System (DAKO, Cat. No. K5007) at room temperature for 30 min. Afterward, the samples were visualized using 3,3'-diaminobenzidine (DAB) chromogen from the REAL EnVision Detection System (DAKO, Cat. No. K5007). Samples were counterstained with hematoxylin, gradually dehydrated using graded alcohol and xylene, and then mounted with mounting medium.

**Table 1:** The list of the primary antibodies for immunohistochemistry.

Molecule	Dilution	Origin	Supplier (catalog no.)
CK8	1/1000 (IHC) 1/100 (IF)	Rabbit monoclonal (EP1628Y)	ABCAM (Cat. #ab53280)
Alpha SMA	1/1000 (IHC)	Rabbit monoclonal (EPR5368)	ABCAM (Cat. #ab124964)
Alpha SMA	1/100 (IF)	Goat polyclonal	ABCAM (Cat. #ab21027)
Ki67	1/500 (IHC)	Rabbit monoclonal (clone SP6)	Funakoshi (Cat. #RM-9106-S1)
CD31	1/300 (IHC)	Rabbit monoclonal (D8V9E)	Cell signaling (Cat. #77699)

### Immunofluorescent staining

Paraffin-embedded samples were sliced at a thickness of 5 μm, deparaffinized in xylene, and rehydrated in a graded alcohol sequence. Antigen retrieval was performed as previously described. Sections were then co-incubated with rabbit anti-CK8 and goat anti-αSMA antibodies (Table 1). Immunofluorescence detection was performed using secondary Alexa Fluor 488 goat anti-rabbit IgG (H + L) pAb (1:200; A-11034; RRID: AB_2576217; Thermo Fisher Scientific, Waltham, MA) and secondary Alexa 568 donkey anti-goat IgG (H + L) pAb (1:200; A-11057; RRID: AB_2534104; Thermo Fisher Scientific) before incubation for 120 min at room temperature. The samples were counterstained with 4′,6-diamidino-2-phenylindole (DAPI). Fluorescent images were obtained using a Zeiss LSM 700 confocal microscope (Carl Zeiss, Germany).

### Measurement of the number and volume of the adenomyosis lesions

**Figure 2:**
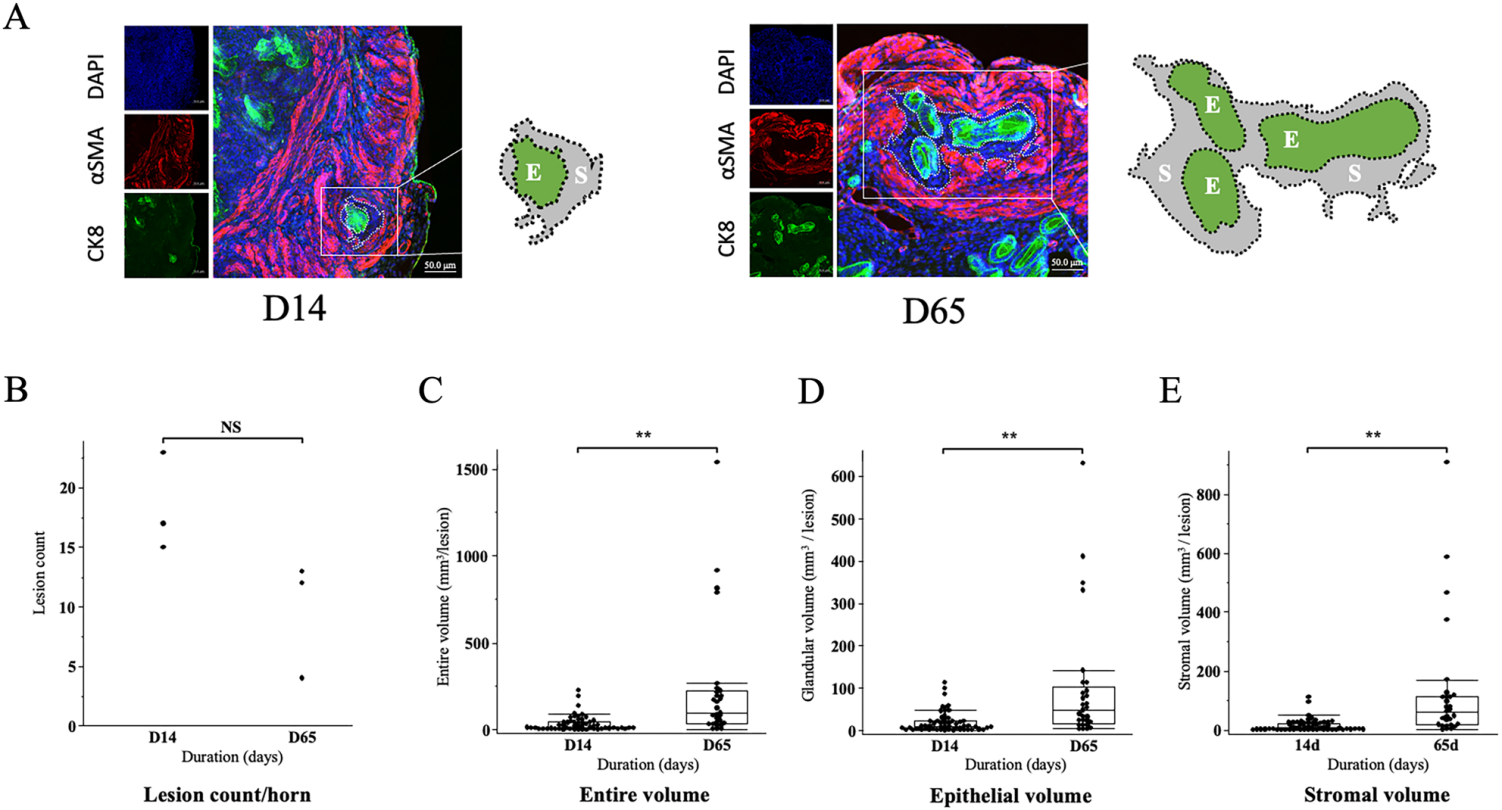
(**A**) A representative immunofluorescence image of the adenomyosis lesion (×400) on the 14th postoperative day (D14) and the 65th postoperative day (D65) with cytokeratin 8 (CK8) (red) and alpha smooth muscle actin (α SMA) (green). In the illustration, the area highlighted in green (E) is the area of the epithelium of the adenomyosis lesion stained with CK8, and the area highlighted in gray (S) is the area of the stroma of the lesion, which is not stained with either aSMA or CK8. (**B**) A scatter plot for the adenomyosis lesion count per horn. Lesion count per horn was compared between the D14 and D65. The count was not significantly different between the groups. (**C–E)** A box and whisker graph for the adenomyosis lesion volume. The lesion volume of the entire (**C**), epithelial (**D**), and stromal (**E**) area was compared between D14 and D65. The cross-sectional area of the lesion was measured by tracing the innermost line of the smooth muscle that was identified by positive staining with αSMA and using it as the outermost line of the lesion. The cross-sectional area of the epithelial part of the lesion was measured by tracing the epithelium, with positive CK8 staining, within the adenomyosis lesion. The cross-sectional area of the stromal part of the lesion was calculated by subtracting the area of the epithelial part from the area of the entire lesion. The volumes of each lesion were calculated as the sum of cross-sectional areas multiplied by the inter-slide spacing. The volume was significantly larger in D65 than in D14 (p < 0.0001). Box: 25–75%, whisker: 10–90%, midline: median, *NS* not significantly different **p < 0.0001.

This study developed a novel method to quantitatively analyze the number and volume of adenomyosis lesions. Once the uterine horn was obtained, the tissues were fixed and embedded in paraffin blocks. The entire uterus was sliced into 6-μm-thick serial sections. On average, 2500 sections were prepared from one horn. All H&E-stained sections were observed, and the number of adenomyosis lesions was counted. The length was calculated by multiplying the total number of slices containing a single lesion (from top to bottom) by 6 μm. The cross-sectional area of the lesion was measured using ImageJ (version 1.53c, National Institutes of Health), by tracing the innermost line of the smooth muscle that was identified by positive staining with αSMA and using it as the outermost line of the lesion (Fig. 2A). The cross-sectional area of the epithelial part of the lesion was measured by identifying regions of epithelium within the adenomyosis lesion with positive CK8 staining (Fig. 2A). The cross-sectional area of the stromal part of the lesion was calculated by subtracting the area of the epithelial part from the area of the entire lesion. The volumes of the lesions were calculated as the sum of the cross-sectional areas multiplied by the inter-slide spacing. The equation used to calculate this is as follows:

$$ {\text{Volume }}\,\left( {{\text{mm}}^{{3}} } \right)\, = \,\sum {\text{ cross}} - {\text{sectional\, area }}\,\left( {{\text{mm}}^{{2}} } \right)\, \times \,0.00{6 }\,\left( {{\text{mm}}} \right). $$

### Evaluation of cell proliferation

Cell proliferation was evaluated by Ki67 IHC. Cells with Ki67-positive nuclei in the epithelial and stromal parts of adenomyosis lesions were counted, and the percentage of cells that were Ki67-positive was calculated.

### Evaluation of vascular density

For vascular density evaluation, CD31, a marker for endothelial cells, was used to detect blood vessels. Blood vessels within the lesions were identified, the cross-sectional area of blood vessels was measured, and this was divided by the cross-sectional area of the adenomyosis lesion to calculate the vascular density.

### Evaluation of fibrosis

Fibrotic regions were identified using Masson’s trichrome staining. The uterine sections were deparaffinized, rehydrated, and transferred to Bouin’s solution at 56 °C for 15 min. Sections were stained using Masson’s Trichrome Staining kit (Sigma-Aldrich Inc., St. Louis, MO) following the manufacturer’s instructions. Images of adenomyosis lesions were randomly taken at a magnification of 40×. The cross-sectional area of fibrotic regions (areas where collagen deposition had occurred) was determined using image analysis software ImageJ (version 1.53c, National Institutes of Health, Bethesda, MD) with the plugin “color deconvolution” for stain separation (MTS1,2). Regions that stained bluer than the automatically calculated threshold were identified as fibrotic, and the cross-sectional areas of these regions were calculated. The percentage of the lesion that was fibrotic was calculated by dividing the area of the fibrotic region by the area of the entire lesion.

### Establishment and evaluation of mouse pregnancy model

To investigate the prognosis of pregnancy in this mouse model and whether the lesions persisted after pregnancy, seven mice were used to model pregnancy. Six-week-old male BALB/c mice were used for mating. Mating was started 7 days after the establishment of adenomyosis and continued for up to 10 consecutive days until the plug was confirmed. Female mice were then kept in a separate cage until delivery, and the date of delivery was recorded. The pups were separated from the dams on the first day after delivery, and litter size was recorded. The dams were sacrificed on the third day after delivery, and the uteri were collected. The number of implantation sites in both horns was determined and recorded. The microscopic appearance of the lesions was observed, and number and volume of adenomyosis lesions were evaluated as described above and compared with those of non-pregnant mice that had been left without mating for the same period of time as the pregnant mice after establishing adenomyosis.

### Statistical analysis

All data analysis were conducted using JMP Pro software (version 15.2.1, SAS Institute Inc., Cary, NC https://www.jmp.com/). All non-parametric data were compared using the Wilcoxon rank sum test. p < 0.05 was considered significant.

### Ethics approval

All animal experiments were approved by the Animal Committee of The University of Tokyo School of Medicine (Approval No. P19-034, 11 July 2019), and the manuscript follows the recommendations of the ARRIVE guidelines.

## Results

### Establishment of the mechanical induced adenomyosis mouse model

For all mice, one horn was punctured, defined as the adenomyosis horn, while the other was left intact, defined as the control horn. The mean time from the start of anesthesia to the end of the procedure was approximately 20 min (data not shown). There were no postoperative complications such as infection or adhesion.

Figure 1B shows the macroscopic appearance of both horns (D14 and D65). At D14, the adenomyosis horn was slightly shorter than the control horn, and the difference became more pronounced in D65, suggesting that the adenomyosis horn was contracted and shortened. Figure 1C demonstrates the microscopic findings of control horns. The endometrial and smooth muscle structure of the adenomyosis horn (Fig. 1D) was similar to that of the control horn (Fig. 1C); however, there was a lesion that formed a glandular tubular structure consisting of epithelium and stroma within the myometrium (Fig. 1D), which resembled human adenomyosis. This "adenomyosis lesion" appeared as early as the 7th postoperative day (Supplemental Fig. S1) and persisted for more than 2 months. There was not a single lesion in the control horn.

### Changes in the number, size of adenomyosis lesions over time

Figure 2A demonstrates an immunofluorescence image of the uterine section with an adenomyosis lesion. As for the lesion count per horn, there was no significant difference between D14 and D65 (Fig. 2B, 18.3 ± 2.4 and 9.7 ± 2.8, D14 and D65, respectively, mean ± SEM, p = 0.08). The volume of the entire lesion was significantly greater in D65 than in D14 (Fig. 2C, D14: 34.5 ± 6.1, D65: 231.5 ± 64.6 mm^3^/lesion, p < 0.0001). The volume of the epithelial part of the lesion was also significantly greater in D65 than in D14 (Fig. 2D, D14: 18.6 ± 3.3, D65: 100.5 ± 27.2 mm^3^/lesion, p < 0.0001). The volume of the stromal part of the lesion was also significantly greater in D65 than in D14 (Fig. 2E, D14:15.8 ± 2.9, D65:131.0 ± 37.9 mm^3^/lesion, p < 0.0001).

### Changes in cell proliferation, vascular density, and fibrosis of adenomyosis lesions over time

**Figure 3:**
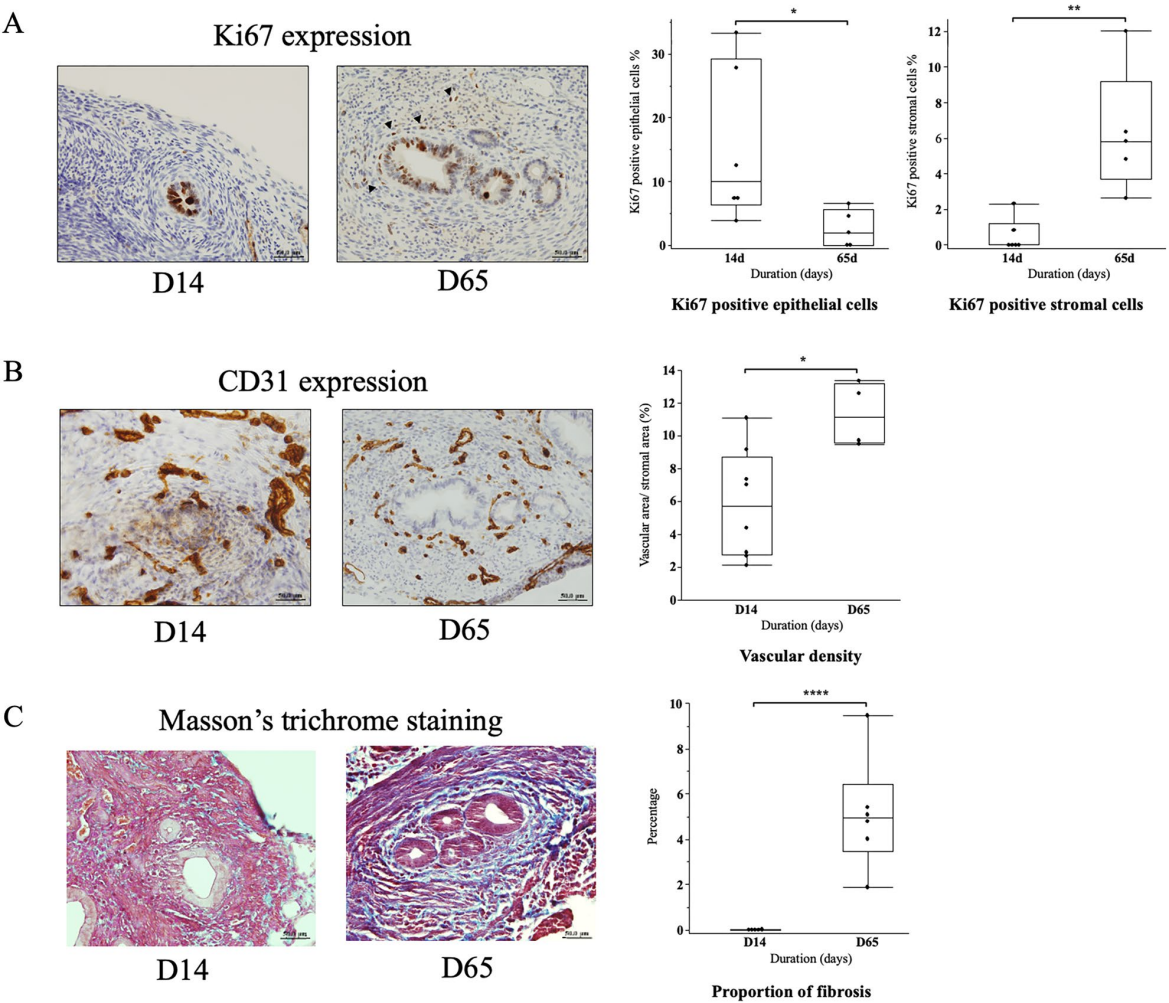
(**A**) A representative microscopic image of Ki67 expression in adenomyosis lesion (× 400) and a box and whisker graph of the percentage of Ki67 positive cells Ki67 is positive in the nuclei of both epithelial and stromal cells (arrowheads) in the adenomyosis lesion at the 14th (D14) (left) and the 65th (D65) (right) postoperative day. In epithelial cells, the percentage of Ki67 positive cells was significantly higher in D14 than in D65 (p < 0.05), while in stromal cells, the percentage was significantly higher in D65 than in D14 (p < 0.01). (**B)** A representative microscopic image of CD31 expression in adenomyosis lesion (×400) and a box and whisker graph of vascular density. CD31, a marker for endothelial cells, was used to detect blood vessels. Blood vessels within the lesion were identified, the cross-sectional area of blood vessels was measured, and this was divided by the cross-sectional area of adenomyosis lesion to calculate the vascular density. Vascular density in D65 was significantly higher than that in D14 (p < 0.05). (**C**) A representative microscopic image of Masson’s trichrome staining of adenomyosis lesion (×400) and a box and whisker graph of percentage of fibrotic area. Areas of fibrosis (collagen deposition) were stained blue. The percentage of fibrosis area in D65 was significantly higher than that in D14 (p < 0.001). Box: 25–75%, whisker: 10–90%, midline: median, *p < 0.05, **p < 0.01, ****p < 0.001.

As shown in Fig. 3A, the percentage of Ki67 positive cells in epithelial part of the lesion was significantly higher in D14 than in D65 (D14: 15.3 ± 5.0%, D65: 2.6 ± 1.3%, p < 0.05), while in the stromal part, the percentage of Ki67 positive cells was significantly higher in D65 than in D14 (D14: 0.5 ± 0.4%, D65: 6.3 ± 1.6%, p < 0.01). As for vascular density (Fig. 3B) the density was significantly higher in D65 than in D14 (D14: 0.06 ± 0.01%, D65: 0.1 ± 0.01%, p < 0.05). The percentage of fibrotic area was significantly higher in D65 than in D14 (D14: 0.007 ± 0.004%, D65: 5.1 ± 1.01%, p < 0.01) (Fig. 3C).

### Evaluation of pregnancy outcome and postpartum lesion in adenomyosis model mice

Five out of seven mice (71.4%) conceived within 10 days of consecutive mating. The date of delivery, the litter size, and the number of implantation sites of each mouse are shown in Table 2. The date of delivery was 19.2 ± 0.8 (mean ± SD) dpc which is slightly earlier than previously reported for the mouse of the same strain (mean ± SD; 20.2 ± 0.1 dpc)^21^ and (mean ± SD; 19.6 ± 0.6 dpc)^22^. The litter size was 4.4 ± 3.4 (mean ± SD), which is smaller than litter size that previously reported in two articles (5.9 ± 2.7^22^ and 5.32 ± 0.2^23^). The number of implantation sites were not different between the control and the adenomyosis horn (control: 2.6 ± 1.8, adenomyosis: 3 ± 1.6, mean ± SD, p = 0.7) (Table 2).

**Table 2:** Adenomyosis pregnancy model.

Mouse	Delivery day (dpc)	Litter size (no.)	Implantation site	
			Control horn	Adenomyosis horn
A	20	2	1	1
B	19	9	5	4
C	18	7	4	5
D	20	3	2	3
E	19	1	1	2
Mean ± SD	19.2 ± 0.8	4.4 ± 3.4	2.6 ± 1.8	3 ± 1.6

**Figure 4:**
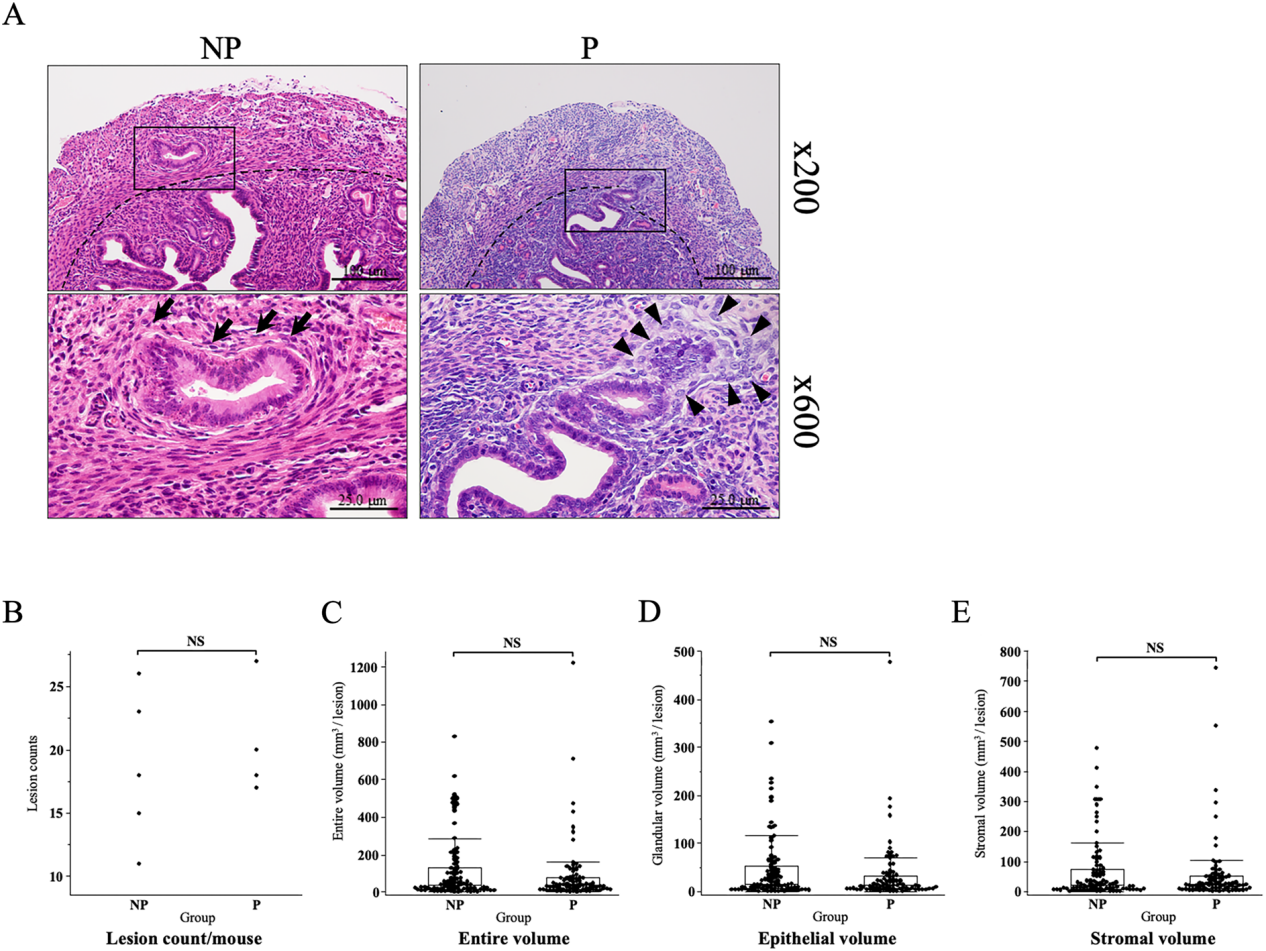
(**A**) A representative microscopic image of the adenomyosis horn (non-pregnant: NP; left, postpartum: P; right) (upper figures; ×200) (lower figures; ×600) hematoxylin and eosin staining of the adenomyosis lesions from non-pregnant (NP) and postpartum (P) mouse. Arrowheads in P mouse indicate a decidualized stromal cells, characterized by the presence of large polygonal cells with a large round or oval vesicular nucleus, while arrows in NP mouse, indicate normal stromal cells, characterized by the presence of spindle shaped cells with a small spindle condensed nucleus. (**B**) A scatters plot for adenomyosis lesion count per mouse. The lesion count in non-pregnant (NP n = 5) and postpartum (P n = 4) mice were compared. (**C–E**) A box and whisker graph for adenomyosis lesion volume. The cross-sectional area of the lesion was measured by tracing the innermost line of the smooth muscle that was identified by positive staining with αSMA and using it as the outermost line of the lesion. The cross-sectional area of the epithelial part of the lesion was measured by tracing the epithelium, with positive CK8 staining, within the adenomyosis lesion. The cross-sectional area of the stromal part of the lesion was calculated by subtracting the area of the epithelial part from the area of the entire lesion. The volumes of each lesion were calculated as the sum of cross-sectional areas multiplied by the inter-slide spacing. The lesion counts (**B**) and volume of the entire (**C**), epithelial (**D**) and stromal (**E**) areas were comparable between NP and P. Box: 25–75%, whisker: 10–90%, midline: median, NS: p > 0.05.

We analyzed histological sections of adenomyosis lesions with hematoxylin and eosin staining. We found that the adenomyosis lesions in postpartum mouse were accompanied by decidualized stromal cells (Fig. 4A arrowheads), while lesions from non-pregnant mouse only showed normal stromal cells (Fig. 4A arrows). We then evaluated the number and the volume of the adenomyosis lesions in the postpartum mouse (n = 4) and compared them with those in non-pregnant mice (n = 5). The lesion count per horn was not different between the groups (non-pregnant: 18.6 ± 2.7, postpartum: 20.5 ± 2.3, mean ± SEM, p = 0.6, Fig. 4B). The volume of the entire lesion was also comparable between the groups (non-pregnant: 109.6 ± 16.5, postpartum: 89.1 ± 18.8 mm^3^/lesion, p = 0.6, Fig. 4C). The volume of the epithelial part of the lesion was also not different between the groups (non-pregnant: 45.1 ± 7.1, postpartum group:31.7 ± 6.9 mm^3^/lesion, p = 0.2, Fig. 4D). The volume of the stromal part of the lesion was also not different between the groups (non-pregnant: 64.5 ± 10, postpartum: 57.5 ± 12.4 mm^3^/lesion, p = 0.9, Fig. 4E).

## Discussion

In this study, we established a novel mouse model of adenomyosis induced by direct mechanical puncture of the uterus. The established lesions had glandular tubular structures consisting of epithelium and stroma within the myometrium, similar to lesions seen in human adenomyosis. We also quantitatively assessed the changes in these lesions over time and found that the lesions did not change in number but increased in size. We also found that, over two months, epithelial cell proliferation decreased, stromal cell proliferation increased, and vascular density and fibrosis increased. In addition, we found that the mice were capable of pregnancy and delivery, and that their lesions persisted after delivery.

This novel adenomyosis model was established by mechanically disrupting the endometrial-myometrial interface. As mentioned in the introduction, several theories have been proposed to explain the etiology of adenomyosis. At least one type of adenomyosis (Type 1 adenomyosis in Kishi’s classification) is known to be more prevalent in women of multiparity and women who have undergone uterine surgery^24–26^. It is widely believed that this type of adenomyosis is caused by mechanical damage to the uterus that disrupts the endometrial-myometrial interface and allows the endometrium to invade the myometrium^19,24,27,28^. In the present mouse model, we attempted to disrupt the endometrial-myometrial interface with repeated punctures that penetrated the entire myometrium. Using this method, we succeeded in generating glandular structures consisting of epithelium and stroma in the myometrium, which resembled human adenomyosis.

One of the advantages of this model is that following uterine puncture, which was a short procedure, the lesion persisted for a long time. Previously described mouse models of adenomyosis take significantly longer to establish. In previous studies, lesions have only been established 10–12 weeks after intervention^20^ or 6 weeks after neonatal tamoxifen administration^15^. In contrast, in our model, lesions are established by day seven at the latest, which has the advantage of allowing us to study drug administration and pregnancy prognosis early after the uterine puncture, while the mice are young. Furthermore, this model was shown to create lesions that persisted for at least 65 days. In contrast, previous studies on mouse models did not examine whether the lesions persisted for a long time, except for a single study by Shen et al.^17^, where lesions were examined on day 60. Thus, this model could be useful for confirming the efficacy of long-term drug treatment.

In the current study, we also established a novel quantitative analysis method for adenomyosis lesions, which involved counting and measuring the volume of lesions using slices of the entire horn. Furthermore, by using markers for epithelial cells and myocytes, we established a method to measure and evaluate the epithelial and stromal parts of the lesion independently. This quantitative analysis allowed a better understanding of temporal changes in lesion growth and regression. The present study revealed that, over time, the number of lesions did not change significantly, and the volume of each lesion significantly increased in both the epithelial and stromal parts. However, it is unclear whether this was due to the fusion of multiple lesions or the regression of some lesions and the growth of others. This is an area that requires further study.

Following on from the previously described finding regarding the increase in lesion size over time, the current study also examined the changes in cell proliferation for both epithelial and stromal compartments over time using Ki67 staining. We found that, in the epithelial compartment, Ki67-positive cells were significantly more abundant in the early stage. In contrast, Ki67-positive cells became significantly more abundant in the stromal compartment as the lesion progressed. This suggests that, in adenomyosis lesions, epithelial cells proliferate first and stromal cells proliferate later, but it is still unclear whether this is due to; epithelial-mesenchymal transition (EMT) or because the epithelial cells cause the attraction and the proliferation of stromal cells by some unknown mechanism. Cell proliferation in eutopic and ectopic endometrium has been studied in endometriosis^29,30^ but not in adenomyosis; therefore, further studies using human specimens and comparisons with the current study should be conducted in the future.

In addition to quantitative assessment, we also qualitatively assessed the characteristics of the lesions, including vessel density and fibrosis. Regarding vascular density, it has been demonstrated that microvessels develop around adenomyosis lesions in the human adenomyosis^31,32^. In the present study, the vascular density progressively and significantly increased over time, which is consistent with findings made in studies using other mouse models of adenomyosis^17,18^. It is possible that the proliferation of these microvessels was responsible for the increase in the volume of adenomyosis lesions over time in our model. It is well known that human adenomyosis lesions are accompanied by fibrosis^33^ due to repeated cyclic bleeding associated with tissue injury and repair^34,35^. In our model, the extent of fibrosis in the adenomyotic lesion significantly increased over time, a finding that is consistent with discoveries made in other studies^17^. The gross findings that the adenomyosis horns were shorter than the control horns may also be due to the contraction of the muscle layer caused by fibrosis. In summary, this model allows quantitative observation of angiogenesis and fibrosis associated with adenomyosis over time and is expected to be useful in determining the efficacy of treatment methods in the future.

Furthermore, we confirmed that these mice were able to conceive and deliver, and that the lesions persisted after delivery. To the best of our knowledge, there have been only two reports^19,36^ that studied the course of pregnancy using an adenomyosis mouse model, but these reports examined the litter size at 30 days after mating but did not evaluate the status of the adenomyosis during the postpartum period. Therefore, the current study is the first one in which mice were allowed to deliver to analyze the status of adenomyosis after delivery. One advantage of our model over the previous model is that it does not require hormonal modification or oophorectomy, which can cause ovarian dysfunction or uterine thinning^13–19^ and may affect the course of pregnancy. This study showed that the number and size of adenomyosis lesions did not change between the pre-pregnancy and post-partum period; therefore, this model may be helpful in modeling perinatal outcomes in pregnancies complicated by adenomyosis. Another interesting finding in the current study is that although the number and size of adenomyosis lesions did not change after pregnancy, pregnancy caused the decidual change in the adenomyosis lesions. Further investigation of the impact of these changes on the pathogenesis of adenomyosis and the perinatal outcomes would provide clinically significant insights.

Finally, our preliminary data on perinatal outcomes showed that adenomyosis mice seemed to have an earlier delivery date and smaller litter size than normal healthy mice of the same strain and age, as reported in previous studies^21–23^. The findings of the small litter size were comparable to those reported in other mouse models of adenomyosis^19,36^. We also found that the number of implantation sites was comparable between the adenomyosis horn and the control horn, suggesting that the presence of adenomyosis not only affects implantation but also affects intrauterine development. These outcomes seem to mimic the high incidence of preterm birth, fetal growth restriction (FGR) and intrauterine fetal death (IUFD).in pregnant women with adenomyosis^9–12^, and this mouse model is expected to be a suitable model for pregnancy complicated by adenomyosis, although further validation is warranted.

One weakness of this study is that although it successfully quantitatively assessed the adenomyosis lesions, it did not evaluate the symptoms or behavior associated with adenomyosis. A quantitative evaluation system such as a hot plate, open field, and tail suspension test would be used to assess symptoms or behavior^37^. Future studies using such methods to examine how adenomyosis or pregnancy affect symptoms or behaviors would be expected to lead to further clinically significant research.

## Conclusions

In this study, we established a mouse model of adenomyosis caused by mechanical damage to the uterus. We showed that the number and volume of lesions could be longitudinally and quantitatively analyzed. Furthermore, we proved that this model could conceive, deliver, and persist after delivery. Collectively, this model can be applied to investigate the pathogenesis of adenomyosis, validate the efficacy of therapeutic agents, and evaluate the effect of adenomyosis on pregnancy and vice versa.

## Supplementary Information

Supplementary Figure S1.

## Data Availability

The datasets used and/or analyzed during the current study are available from the corresponding author upon reasonable request.

## Abbreviations

αSMA: Alpha smooth muscle actin
CK8: Cytokeratin 8
D14: Day 14
D65: Day 65
DAPI: 4’,6-Diamidino-2-phenylindole
H&E: Hematoxylin and eosin
HMB: Heavy menstrual bleeding
IHC: Immunohistochemistry
EMT: Epithelial–mesenchymal transition
FGR: Fetal growth restriction
IUFD: Intrauterine fetal death
