# Supplementary material for: Establishment of a novel mouse model of adenomyosis suitable for longitudinal and quantitative analysis and perinatal outcome studies
Source: Sci Rep. 2022 Oct 20;12:17515. doi: 10.1038/s41598-022-22413-8 (PMC9585053; doi:10.1038/s41598-022-22413-8)
Supplement: Supplementary file 1 — Supplementary Figure S1. [file 41598_2022_22413_MOESM1_ESM.docx]

**
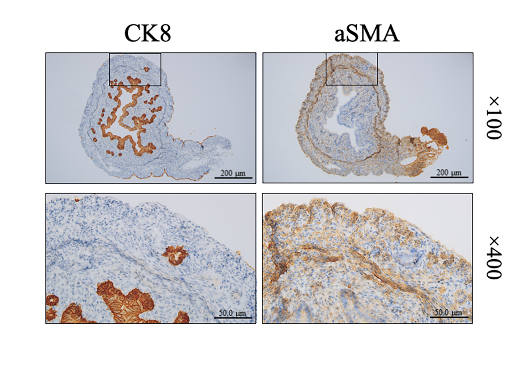
**

**Supplementary Fig. S1 A representative microscopic image of the adenomyosis horn (D7, upper figures; ×100) (lower figures; ×400)**  Cytokeratin 8 (CK8) shows eutopic endometrial epithelium and epithelium of the lesion and (marked with black box), alpha smooth muscle actin (α SMA) shows smooth muscle structure that encircles adenomyosis lesion (marked with black box). It can be observed that the epithelium has already penetrated into the muscle layer.
